# Prevalence of burnout in paediatric nurses: A systematic review and meta-analysis

**DOI:** 10.1371/journal.pone.0195039

**Published:** 2018-04-25

**Authors:** Laura Pradas-Hernández, Tania Ariza, José Luis Gómez-Urquiza, Luis Albendín-García, Emilia I. De la Fuente, Guillermo A. Cañadas-De la Fuente

**Affiliations:** 1 University Hospital Health Technology Park, Andalusian Health Service, Granada, Spain; 2 Department of Educational Psychology and Psychobiology, Faculty of Education, International University of La Rioja, La Rioja, Spain; 3 Department of Nursing, Faculty of Health Sciences, University of Granada, Ceuta, Spain; 4 South Cordoba Health Management Area, Andalusian Health Service, Cordoba, Spain; 5 Department of Nursing, Faculty of Health Sciences, University of Granada, Granada, Spain; Mohammed bin Rashid University of Medicine and Health Sciences, UNITED ARAB EMIRATES

## Abstract

**Introduction:**

Although burnout in paediatric nurses has been addressed in previous research, the heterogeneous nature of the results obtained and of the variables studied highlights the need for a detailed analysis of the literature.

**Objective:**

The aim of this study was to analyse the literature on burnout characteristics, reported prevalence, severity and risk factors, to achieve a better understanding of the risk of emotional exhaustion, depersonalisation and feelings of low personal accomplishment.

**Method:**

For this purpose, we carried out a systematic review and meta-analysis of the literature. The databases consulted were CINAHL, LILACS, PubMed, the Proquest Platform (Proquest Health & Medical Complete), Scielo and Scopus. This study used the search equation “burnout AND “pediatric nurs*””, and was conducted in July 2017.

**Results:**

The search produced 34 studies targeting burnout in paediatric nurses, with no restrictions on the date of publication. Many of these studies detected moderate-high values for the three dimensions of burnout, and highlighted sociodemographic, psychological and job-related variables associated with this syndrome. The sample population for the meta-analysis was composed of 1600 paediatric nurses. The following prevalence values were obtained: (i) emotional exhaustion, 31% (95% CI: 25–37%); (ii) depersonalisation, 21% (95% CI: 11–33%); (iii) low personal accomplishment, 39% (95% CI: 28–50%).

**Conclusions:**

A significant number of paediatric nurses were found to have moderate-high levels of emotional exhaustion and depersonalisation, and low levels of personal accomplishment. These nurses, therefore, were either experiencing burnout or at high risk of suffering it in the future. These results support the need for further study of the risk factors for burnout in paediatric nurses. They also highlight the importance of developing interventions or therapies to help prevent or attenuate the above symptoms, thus helping nurses cope with the workplace environment and with situations that may lead to burnout.

## Introduction

In recent years, the question of occupational burnout has caught the attention of researchers and professionals [1], who have used various instruments to analyse and measure the factors associated with this condition [2–11]. Burnout syndrome among paediatric nurses is characterised by the following effects: (i) emotional exhaustion (EE) with progressive loss of energy; (ii) depersonalisation (D), reflected in negative attitudes towards patients and co-workers; (iii) feelings of low personal accomplishment (PA) or loss of confidence [12, 13]. Workers may be continuously subjected to stressors both at work and in the home. The syndrome is more prevalent in service professions such as teaching, the police and health care [14].

Healthcare professionals, especially nurses, are a high-risk group that is especially prone to the syndrome [15, 16]. The nursing profession demands high levels of social responsibility, and problems that can arise on a daily basis include work overload, lack of autonomy or authority to make decisions, and difficulty in reconciling family life and work. All of these factors can trigger burnout syndrome, generating symptoms such as fatigue, memory problems, depression, anxiety, sleep disorders, irritability or substance abuse [17]. Other stressful situations that nurses must routinely cope with include obligatory rotating shifts, work overload because of understaffing and, in certain cases, care of the terminally ill [17].

All of these factors make nursing professionals particularly vulnerable to burnout. When nurses experience this syndrome, the healthcare institution is also negatively affected because of increased workplace absenteeism and nursing turnover [18,19]. The resulting deterioration in the quality of healthcare is, in turn, detrimental to the users of healthcare services [20].

There is a high prevalence of burnout among nursing professionals [21]. Nurses working in oncology and critical care units are generally regarded as being most susceptible to developing this syndrome [15, 22–24]. However, those in paediatric and neonatal units are also subject to high levels of stress and are at risk of burnout [3, 25, 26]. Detailed knowledge of the variables related to this syndrome, an estimation of the prevalence of each of its dimensions, and an understanding of the context in which these professionals work are all essential elements in the design and implementation of strategies for the treatment and prevention of burnout. Appropriate strategies in this regard would help improve the occupational health of paediatric nurses and the quality of care received by their patients and also contribute to optimising work conditions [27, 28].

In summary, the aim of this study was to determine burnout prevalence, levels and risk factors in paediatric nurses and to conduct a meta-analysis to estimate the prevalence of emotional exhaustion and depersonalisation and of feelings of inadequate personal accomplishment.

## Materials and methods

### Data sources, search equation and inclusion criteria

A systematic review and meta-analysis were performed according to the PRISMA guidelines [29]. The first step in this process was to search the following electronic databases: CINAHL, LILACS, PubMed, the Proquest Platform (Proquest Health & Medical Complete), Scielo and Scopus. The search equation used was “burnout AND “pediatric nurs*”. To minimise publication bias, no restrictions were placed on publication date, study methods, language or sample size. The databases also included grey literature. The second step was to review all the previous meta-analytic studies and systematic reviews of this topic. Finally, all the references included in the selected studies were also reviewed.

The following inclusion criteria were applied: (a) primary quantitative studies; (b) studies that assessed burnout risk factors, measured burnout in the sample or specified the number or proportion of subjects with burnout; (c) sample of paediatric or neonatal nurses; (d) language used in the paper (Spanish, English, Portuguese or Italian); (e) studies using the Maslach Burnout Inventory (MBI) were included in the meta-analysis.

The exclusion criteria were: (a) sample of paediatric resident nurses; (b) studies with mixed samples that did not provide independent information for paediatric or neonatal nurses.

### Codification of results and data analysis

The variables were recorded on a data definition log. Two members of the research team independently performed the search, selection and detailed reading of the publications. In case of disagreement, a third researcher was consulted. The following variables were considered:

*Publication variables*: (a) authors; b) year of publication; (c) country of publication; (d) language (Spanish, English, Portuguese or Italian); (e) sample size; (f) percentage of female nurses in the sample; (g) age in years of the sample population (mean, standard deviation, median or range).

*Methodological variables*: (h) burnout measurement instrument; (i) in the case of the MBI, specification of subtype (MBI, MBI-HSS, MBI-GS); (j) use of original instrument or an adapted version; (k) reliability index, either calculated or derived from other studies; (l) estimated reliability coefficient of the instrument.

*Burnout measurement variables*: main results for the presence of burnout in paediatric nurses, including: (i) the prevalence of low, medium and high levels of each burnout dimension (emotional exhaustion, depersonalisation and personal accomplishment); (ii) mean value and standard deviation of the score for each burnout dimension; (iii) prevalence of nursing professionals with burnout.

### Data analysis

A descriptive analysis was made of the study variables included, reporting on the quality of the publications selected, based on the levels of evidence and grades of recommendation proposed by the Oxford Centre for Evidence-Based Medicine (OCEBM) Levels of Evidence Working Group [30].

A sensitivity analysis was also performed to ascertain whether any of the studies in the meta-analysis produced a variation in the estimate of the mean effect size. Publication bias, which refers to the higher probability of studies with statistically significant results being published, was assessed by Egger’s linear regression test. The *Q* test and the *I*^*2*^ index were used to evaluate the heterogeneity that is not explained by the chance. The analyses were performed using StatsDirect Version 2.8.0 (2013), a statistical software package for general health science users.

## Results

### Description of the search and the studies included

**Fig 1:**
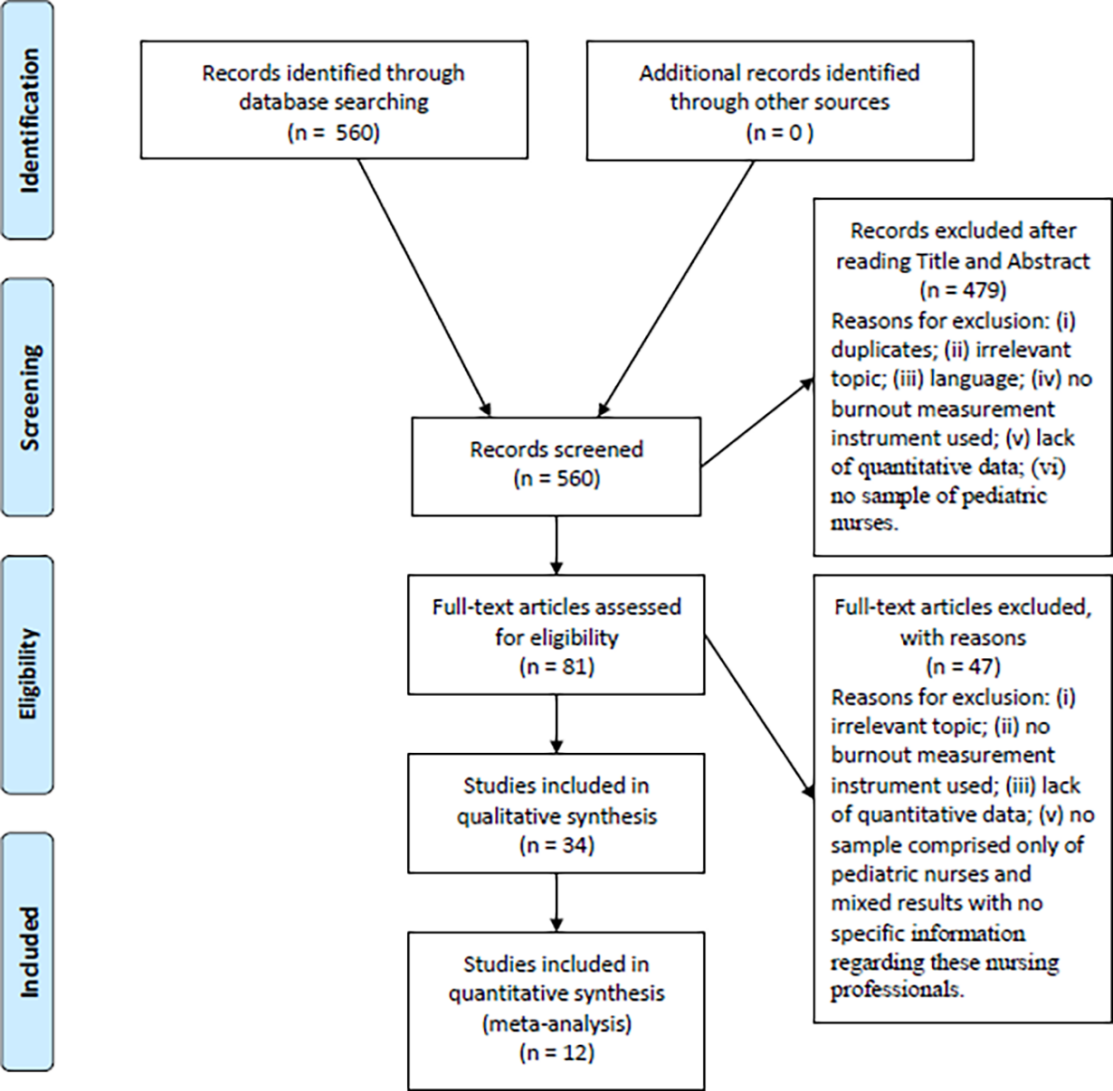
Flow diagram of the publication search process.

The search was performed in July 2017. After applying the corresponding inclusion and exclusion criteria, a total of 34 studies were selected. The sample population was composed of 9,075 paediatric and neonatal nurses (Fig 1).

Almost half of the studies included were published from 2013 to 2016 [2, 5, 31–40] and over 70% were from the US or Canada. Other studies reflected work carried out in Brazil, Egypt, Greece, Pakistan, Peru, Taiwan, Turkey and Venezuela (Table 1).

**Table 1:** Main results of the studies included in the review.

Authors (publication year), country.	Burnout instrument (Instrument Reliability, Cronbach Alpha)	n	Gender and mean age	Burnout-related variables	OCBEM ^a^
Adwan (2014) [31], USA.	MBI-HSS	120	95% Female Age = 34(10) Min-max: 23−62	High levels of grief correlate positively with EE	LE: 2C GE: B
Akman et al., (2016) [52], Turkey.	MBI-HSS	165	Age = 28.95(6)	Job satisfaction, marital status, age and number of assigned patients are all related to burnout. Young, married nurses who work the day shift are most vulnerable to burnout.	LE: 2C GE: B
Alves & Guirardello (2016) [32], Brazil.	MBI-HSS (EE = .87)	267	91.8% Female Age = 34.9 (7.9)	High levels of EE correlate with low levels of job satisfaction and patient safety	LE: 2C GE: B
Aytekin et al., (2013) [51] Turkey.	MBI-HSS (EE = .78, D = .55, PA = .76)	80	Age = 22−29: 37.5% 30−35: 36.2%	PA increases with the nurses’ age and the number of years that they have worked in the paediatric unit. Their quality of life worsens as burnout becomes more intense.	LE: 2C GE: B
Battles (2000) [53], USA.	MBI-HSS (EE = .90, D = .78, PA = .76)	148	98.6% Female Age = 40.3(8.9)	Burnout levels are lower in nurses who spend most of their time in direct contact with the patient, who have fewer patients under their care, and who participate in clinical studies.	LE: 2C GE: B
Berger et al., (2015) [2], USA.	Professional Quality of Life Scale, Version 5	239	97.9% Female Age = >40 (52.1%)	Younger nurses are more prone to burnout	LE: 2C GE: B
Berkowitz (1993) [58], USA & Puerto Rico.	MBI-HSS (EE = .91, D = .75, PA = .78)	518	95% Female	The variables, refusal to care for the patient, the feeling of uselessness, fear and anxiety all correlate positively with EE and D, and negatively with PA.	LE: 2C GE: B
Bilal & Ahmed (2016) [33], Pakistan.	MBI-HSS (EE = .75)	113	72% Female Age = 43.56	High EE correlates negatively with participation in decision-making, communication skills and opportunities for promotion.	LE: 2C GE: B
Budnik (2003) [57], USA.	MBI-HSS	20	-	Emotional intelligence correlates negatively with EE and D, and positively with PA.	LE: 2C GE: B
Czaja et al., (2012) [20], USA.	MBI-HSS	173	93% Female Age: 35(9)	Burnout is associated with the idea of changing job/profession.	LE: 2C GE: B
Davis et al., (2013) [34], USA.	MBI-HSS	15	-	EE correlates negatively with job satisfaction and positively with the nurses’ age.	LE: 2C GE: B
Downey et al., (1995) [3], USA.	Adaptation of Popoff and Funkhouser´s survey of nurses	59	58 Females	Burnout is positively associated with stress.	LE: 2C GE: B
Edmonds et al., (2012) [59], USA.	MBI-HSS	88	-	Burnout is associated with the nurses’ age.	LE: 2C GE: B
Estabrooks et al., (2011) [27], Canada.	MBI-GS	844	-	EE is negatively related to job satisfaction.	LE: 2C GE: B
Gallagher & Gormley (2009) [17], USA.	MBI-HSS	30	-	Length of experience in the profession and working in the medical unit both correlate negatively with EE and D, and positively with PA.	LE: 2C GE: B
Hawes (2009) [56], USA.	MBI-HSS (EE = .90, D = .56, PA = .83)	75	98.7% Female	Stress correlates positively with EE and D, and negatively with PA. The work environment is negatively related to EE and D, and positively to PA.	LE: 2C GE: B
Jacobs et al., (2012) [4], USA.	MBI-HSS & Copenhagen Burnout Inventory	47	-	Gender correlates with burnout.	LE: 2C GE: B
Li et al., (2014) [5], USA.	Compassion Satisfaction Burnout Inventory (Burnout subscale: α = .90)	251	231 Females	Nurses with higher levels of stress present higher levels of burnout.	LE: 2C GE: B
Liakopoulou et al., (2008) [24], Greece.	MBI-HSS (EE = .80, D = .60, PA = .67)	37	-	Burnout is positively associated with being childless, having less job experience, and lacking a clear professional role.	LE: 2C GE: B
Lin et al., (2016) [6], Taiwan.	Occupational Burnout Inventory (Burnout = .94)	144	100% Female Age = 35.72 (7.10)	There is a positive association between stress, burnout and depression.	LE: 2C GE: B
Messmer et al., (2011) [41], USA.	MBI-HSS (α = .90)	33	82% Female Age = 21−27(76%)	Burnout correlates negatively with job satisfaction.	LE: 2C GE: B
Meyer et al., (2015) [8], USA.	Compassion Fatigue Self-Test (Burnout subscale α = .90)	251	231 Females Age = Between 23−30 years (60.1%)	Stress is positively related to burnout in paediatric nurses.	LE: 2C GE: B
Moussa & Mahmood (2013) [54], Egypt.	MBI-HSS	55	Age = 30.3 (7.4)	Age, length of professional experience, and experience in the medical unit all correlate negatively with EE and D, and positively with PA. Lack of work information correlates positively with EE.	LE: 2C GE: B
Oehler et al., (1991) [43], USA.	MBI-HSS	49	100% Female Age = 29.9	Burnout is related to higher levels of work stress and anxiety, the perceived lack of supervision, and less work experience.	LE: 2C GE: B
Oehler & Davidson (1992) [42], USA.	MBI-HSS	121	100% Female Age = 30.51	Burnout is related to professional experience. Nurses with less experience present higher levels of burnout.	LE: 2C GE: B
Pagel & Wittmann (1986) [9], USA.	Tedium Measure	74	100% Female	There is a positive association between burnout and caring for children with behaviour problems.	LE: 2C GE: B
Parada et al., (2005) [55], Venezuela.	MBI-HSS	104	93.3% Female Age = >40(56.73%)	Burnout levels are higher among younger nurses with less work experience, and among those who spend more time caring for patients.	LE: 2C GE: B
Sekol & Kim (2014) [35], USA.	Professional Quality of Life Scale version V (ProQOL-V) (Burnout subscale: α = .75)	240	94.2% Female	Nurses in a unit with a special quality of life and care programme for patients have lower levels of burnout.	LE: 2C GE: B
Shoffner (1988) [10], USA.	Staff Burnout Scale for Health Professionals (α = .93)	18	-	Nurses on the day shift and those on 12-hour shifts have higher levels of burnout.	LE: 2C GE: B
Squires et al., (2013) [36], Canada.	MBI-GS	735	94.1% Female Age = 20−29(34.1%)	The level of depersonalisation is negatively correlated with the application of research information in the work context.	LE: 2C GE: B
Stimpfel et al., (2013) [37], USA.	MBI-HSS	3710	Age = 44 Nearly all nurses were female	-	LE: 2C GR: B
Vásquez-Manrique et al., (2014) [38], Peru.	MBI-HSS	16	-	Depersonalisation is positively related to the nurse’s job experience and whether their spouse is in employment. Personal accomplishment is correlated with gender and type of contract.	LE: 2C GR: B
Wilkinson (2014) [39], Canada.	MBI-HSS (EE = .91, D = .73, PA = .81)	171	96% Female Age = 26−60	Nurses’ resilience is negatively correlated with levels of emotional exhaustion and depersonalisation, and positively so with PA.	LE: 2C GR: B
Zanatta & Lucca (2015) [40], Brazil.	MBI-HSS	65	-	Burnout is related to being married and to the presence of occupational health problems.	LE: 2C GR: B

EE [Emotional Exhaustion]; D [Depersonalisation]; PA [Personal Accomplishment].

LE [Level of evidence]; GR [Grade of recommendation].

^a^OCEBM [Levels of evidence of the Oxford Centre for Evidence-Based Medicine].

The nurses studied worked in the following areas: paediatrics (*n* = 27 studies), neonatal (*n* = 6) and both services (*n* = 1). Most of these nurses were female, in proportions ranging from 82% [41] to 100% [42, 43]. The ages of the nurses ranged from 23 to 62 years, but 60% were aged 23–30 years [8]. Over 75% of the studies included used the MBI to measure burnout [44–48] (Table 1).

Reliability coefficients of the burnout questionnaires were estimated in 14 of the studies, while the others used reliability induction [49, 50]. The reliability estimates ranged from 0.55 [51] to 0.94 [6], with values higher than 0.7 being considered acceptable (Table 1).

### Burnout risk factors in paediatric services

The development of burnout in paediatric nurses may be influenced by a wide variety of psychological, occupational and sociodemographic factors [23].

*Sociodemographic factors*: Few studies have analysed the relation between nurses’ age and marital status and burnout. According to the publications analysed, married status is associated with lower levels of burnout [52], while age is inversely related to burnout levels, with younger nurses being more prone to this condition [2, 39, 52], although one study [34] reported a positive correlation between age and EE.

*Occupational factors*: Various occupational factors are related to the development of burnout syndrome. Some reports observe that high levels of job satisfaction are correlated with low burnout in paediatric nurses [52, 35]. A negative correlation has also been recorded between EE and job satisfaction [34].

Another factor identified as a burnout risk factor is that of the length of the working day. Nurses with a workday longer than eight hours are reported to have higher levels of job dissatisfaction and burnout than those with a shorter working day [37]. The number of patients assigned to each nurse, too, is directly related to burnout levels; thus, the more patients the nurses must attend, the higher the level of burnout [52, 53]. Finally, factors such as greater difficulty in accessing information at the workplace and less professional experience also make nurses more vulnerable to burnout syndrome [42, 54, 55].

*Psychological factors*. Exposure to stress and anxiety, lack of emotional intelligence and a perceived lack of supervision are associated with higher levels of burnout [8, 42, 56–58], as is a poorer quality of life [51].

### Prevalence and levels of burnout in paediatric nursing

The studies considered estimated burnout, measured as the mean score and standard deviation, both as a total measure and for the subscales, and also included the level of burnout (low, medium, or high). The mean scores varied considerably, in all three dimensions of burnout. Thus, the emotional exhaustion scores ranged from 9.64 to 30.25 [56, 57], the depersonalisation values from 3.37 to 7.43 [56, 57] and those for personal accomplishment from 10.94 to 40.21 [54, 55] (Table 1).

Large variations were also observed in the prevalence of burnout symptoms. Thus, the prevalence of high levels of emotional exhaustion ranged from 12.5 to 56.4. [38, 54], and of depersonalisation, from 5.5 to 74.5 [53, 55], while the prevalence of low levels of personal accomplishment ranged from 6.67 to 85.5 [34, 54] (Table 2).

**Table 2:** Prevalence of high EE, high D and low PA.

Authors (publication year), country	n	High EE	High D	Low PA
Alves & Guirardello (2016) [32], Brazil.	267	27.3%	-	-
Battles (2000) [53], USA.	148	31.7%	5.5%	50.3%
Berkowitz (1993) [58], USA & Puerto Rico.	518	24%	10%	51%
Czaja et al., (2012) [20], USA.	173	45%	38%	46%
Davis et al., (2013) [34], USA.	15	-	-	6.67%
Edmonds et al., (2012) [59], USA.	88	34.1%	25%	30.7%
Gallagher & Gormley (2009) [17], USA.	30	26.7%	3.3%	16.7%
Moussa & Mahmood (2013) [54], Egypt.	55	56.4%	74.5%	14.5%
Oehler & Davidson (1992) [42], USA.	121	32.7%	11.9%	36.6%
Parada et al., (2005) [55], Venezuela.	104	16.35%	22.12%	13.46%
Vásquez-Manrique et al., (2014) [38], Peru.	16	12.5%	12.5%	62.5%
Zanatta & Lucca (2015) [40], Brazil.	65	24.6%	29.8%	22.8%

EE [Emotional Exhaustion]; D [Depersonalisation]; PA [Personal Accomplishment].

### Results of the meta-analysis

The sensitivity analysis, conducted to determine whether any of the studies influenced the overall result, revealed no statistically significant changes in burnout prevalence when each study in turn was excluded from the analysis. Nor did the assessment of publication bias detect any statistically significant results.

The following results were obtained by the Cochran’s *Q* test: emotional exhaustion, 58.73 (*p*<0.001); depersonalisation, 186.64 (*p*<0.001); personal accomplishment, 146.19 (*p*<0.001). The *I*^*2*^ was indicative of a high level of heterogeneity with values of 83% for emotional exhaustion; 95.2% for depersonalisation; and 93.2% for personal accomplishment.

**Fig 2:**
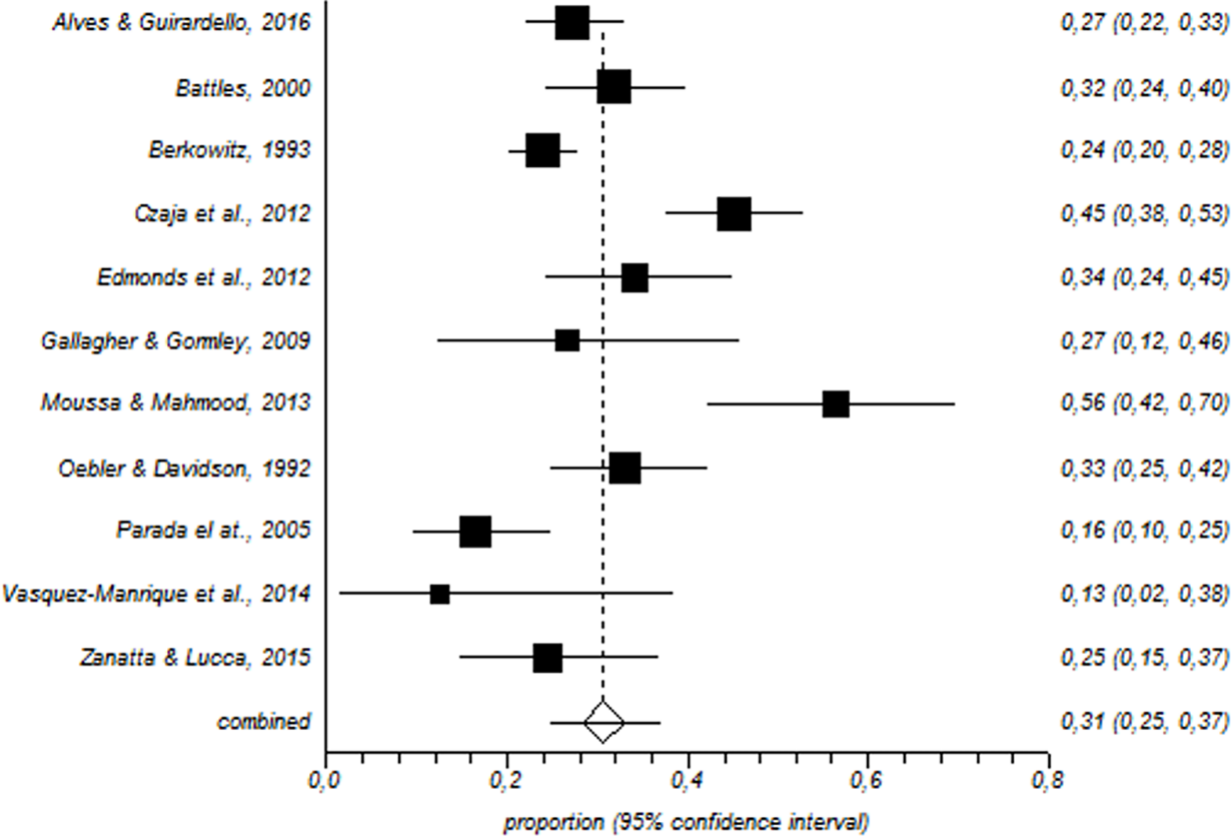
Forest plot for high emotional exhaustion.

**Fig 3:**
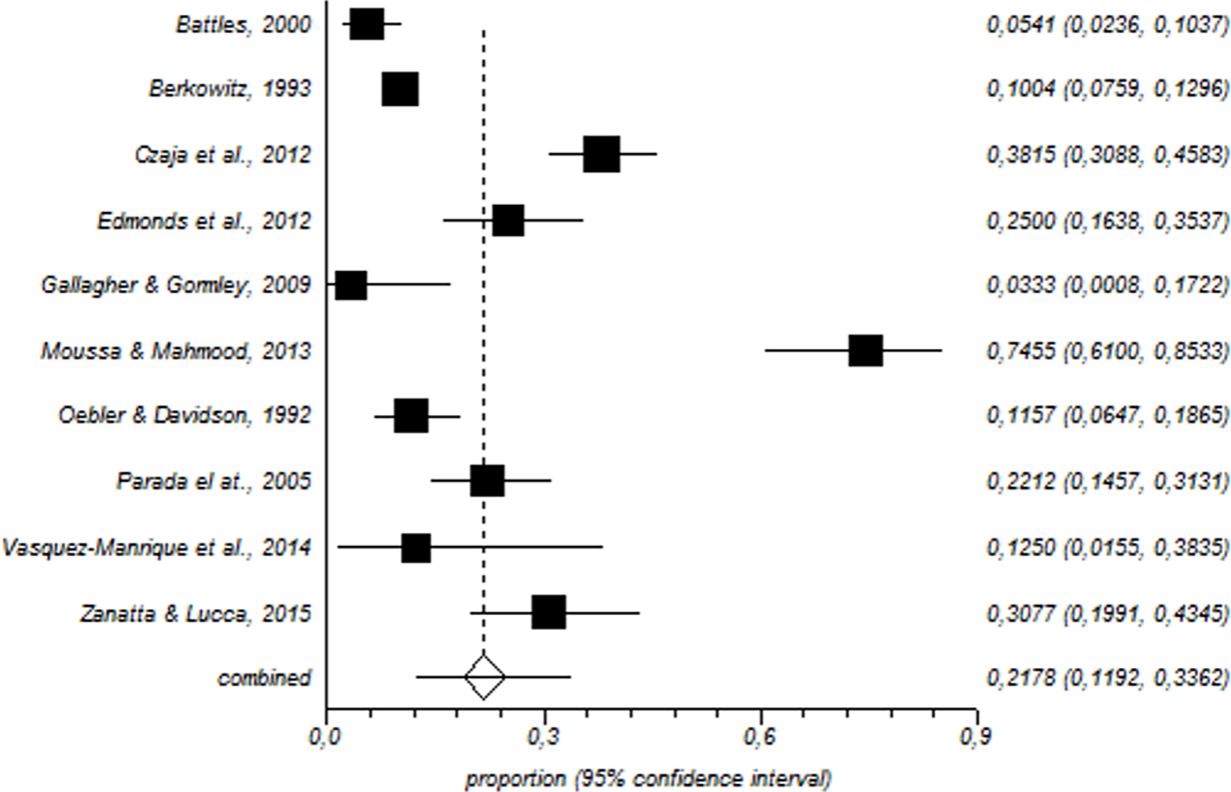
Forest plot for high depersonalisation.

**Fig 4:**
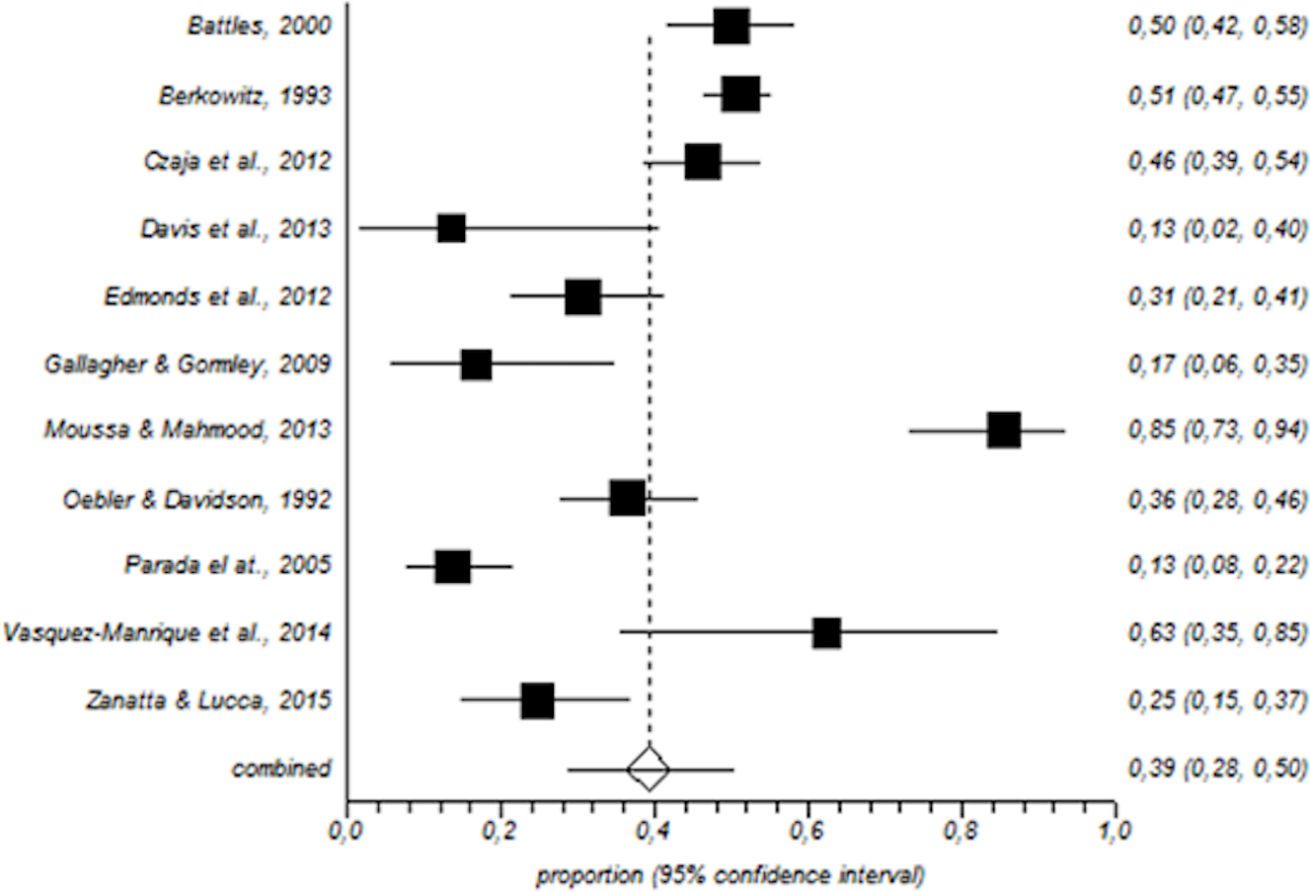
Forest plot for low personal accomplishment.

In total, 1600 paediatric nurses were included in the meta-analysis. Among this population, the prevalence of a high level of emotional exhaustion was 31% (95% CI = 25–37%) (Fig 2); for depersonalisation, the corresponding values were 21% (95% CI = 11–33%) (Fig 3) and for low personal accomplishment, 39% (95% CI = 28–50%) (Fig 4).

### Burnout prevention measures

Some studies assessed the effectiveness of different interventions for reducing burnout. Thus, Edmonds et al. [59] reported that the *Care for the Professional Caregiver Program* (including confidentiality discussion, presentation of the model of vicarious trauma and loss, hands-on stress reduction techniques, etc.) was effective in reducing emotional exhaustion [59]. Other authors informed that head nurses should foment group cohesion, seeking to reduce burnout levels [5].

Adwan recommended the implementation and evaluation of intervention programmes [31]. According to Mousa & Mahmood [54], there is an urgent need to establish educational programmes to improve the communication skills of paediatric nurses, which would contribute to reducing burnout. Other authors have concluded that burnout levels could be reduced by restructuring and facilitating the administrative work performed by nurses [53].

## Discussion

To our knowledge, this is the first meta-analysis focused on burnout syndrome in paediatric nurses. This analysis revealed a prevalence ranging from 30.77% to 73.70% for EE, from 26.20% to 73.70% for depersonalisation and from 46% to 85.50% for low PA.

Similar values for emotional exhaustion have been identified in oncology nurses [60], while emergency nurses have shown higher levels of emotional exhaustion [61]. The prevalence of depersonalisation is 6% lower in oncology nurses [60], and 15% higher in emergency nurses [61]. However, compared to both these groups, paediatric nurses present a higher prevalence of low personal accomplishment.

The results obtained are subject to variability in methodological factors such as sample size, measurement instrument used and application of reliability induction. The studies based on larger samples reported less extreme burnout scores, possibly due to the non-probabilistic nature of the sample and/or biases that may have affected the results.

Although we cannot affirm that the country in which the study was carried out is a key factor in burnout levels, this variable should be taken into account, because the characteristics of the health system in question and the culture and language of the country may influence the prevalence and severity of the syndrome. For example, although the sample was small, the study carried out in Egypt recorded high levels of burnout. The interpretation and extrapolation of these results should take into account aspects such as the validation of the measurement instrument, the understanding of the concept of burnout in Egypt, and the working conditions of paediatric nurses in that country [54].

The burnout measurement instrument and the psychometric data included in the research that we reviewed were also subject to some variability; the most frequently used scale was the Maslach Burnout Inventory (MBI), but many authors used alternative measures. Relatively few studies published reliability coefficients of the measuring instruments, although these data are very important to facilitate the generalisation of the results obtained. Some authors performed a reliability induction in their studies, which in certain cases could be deceptive [49, 50, 62].

## Conclusions

A significant number of the paediatric nurses in the sample were found to have medium or high levels of emotional exhaustion and depersonalisation, together with low levels of personal accomplishment. This caused them to suffer burnout or placed them at high risk of developing the syndrome. Certain sociodemographic variables (age), work variables (job satisfaction and workday duration) and psychological variables (stress) should be taken into account in designing measures to prevent the development of burnout syndrome.

Other factors to be considered in evaluating studies of burnout (as well as the interpretation of the results and their possible application to the professional field) include sample size and selection, the adaptation/validation of the measurement instruments used, whether a reliability induction was performed, the health system in the country where the study was carried out, and how the people in that country understand the concept. Finally, other areas such as burnout among paediatricians, and interventions aimed at preventing burnout, directed at all those working in paediatric units, should also be assessed.

### Implications for nursing management

This systematic review contributes valuable information concerning burnout among paediatric nurses. it is highly relevant to the scientific community as a whole, but particularly to workers in public health systems. The results obtained show that the prevalence of this syndrome is medium to high, according to the burnout dimension considered, which suggests that a significant number of paediatric nurses are either affected by burnout or liable to develop it in the near future.

In view of the findings presented in this review, further longitudinal studies should be conducted, focusing on burnout risk factors, measures to attenuate the symptoms observed and coping strategies. A better understanding of these questions would significantly contribute to preventing burnout syndrome among paediatric nurses.

### Policy and practice implications

The results we present show that interventions should be undertaken to reduce the exhaustion that affects many paediatric nurses and to strengthen their resilience regarding the work, patients and co-workers. Nursing supervisors and managers need to be more aware of the fact that working in a paediatric unit can provoke burnout among nurses. Thus, providing better workplace conditions [61], and the introduction of interventions like mindfulness [63] or support groups where nurses can talk about their feelings [60], can have a positive impact in alleviating this syndrome. If this can be achieved, it will have a positive effect not only on the nurses but also on the quality of care provided and on patient satisfaction.

## Supporting information

S1 File Preferred Reporting Items for Systematic Reviews and Meta-Analyses (PRISMA) checklist. (PDF)

S2 File Meta-analysis minimal dataset. (XLSX)
